# Proteomic analysis identifies interleukin 11 regulated plasma membrane proteins in human endometrial epithelial cells *in vitro*

**DOI:** 10.1186/1477-7827-9-73

**Published:** 2011-05-30

**Authors:** Joanne Yap, Caroline FH Foo, Ming Yee Lee, Peter G Stanton, Evdokia Dimitriadis

**Affiliations:** 1 Prince Henry's Institute of Medical Research, Clayton VIC, 3168, Australia

## Abstract

**Background:**

During the peri-implantation period, the embryo adheres to an adequately prepared or receptive endometrial surface epithelium. Abnormal embryo adhesion to the endometrium results in embryo implantation failure and infertility. Endometrial epithelial cell plasma membrane proteins critical in regulating adhesion may potentially be infertility biomarkers or targets for treating infertility. Interleukin (IL) 11 regulates human endometrial epithelial cells (hEEC) adhesion. Its production is abnormal in women with infertility. The objective of the study was to identify IL11 regulated plasma membrane proteins in hEEC *in vitro *using a proteomic approach.

**Methods:**

Using a 2D-differential in-gel electrophoresis (DIGE) electrophoresis combined with LCMS/MS mass spectrometry approach, we identified 20 unique plasma membrane proteins differentially regulated by IL11 in ECC-1 cells, a hEEC derived cell line. Two IL11 regulated proteins with known roles in cell adhesion, annexin A2 (ANXA2) and flotillin-1 (FLOT1), were validated by Western blot and immunocytochemistry in hEEC lines (ECC-1 and an additional cell line, Ishikawa) and primary hEEC. Flotilin-1 was further validated by immunohistochemistry in human endometrium throughout the menstrual cycle (*n = 6-8/cycle*).

**Results:**

2D-DIGE analysis identified 4 spots that were significantly different between control and IL11 treated group. Of these 4 spots, there were 20 proteins that were identified with LCMS/MS. Two proteins; ANXA2 and FLOT1 were chosen for further analyses and have found to be significantly up-regulated following IL11 treatment. Western blot analysis showed a 2-fold and a 2.5-fold increase of ANXA2 in hEEC membrane fraction of ECC-1 and Ishikawa cells respectively. Similarly, a 1.8-fold and a 2.3/2.4-fold increase was also observed for FLOT1 in hEEC membrane fraction of ECC-1 and Ishikawa cells respectively. *In vitro*, IL11 induced stronger ANXA2 expression on cell surface of primary hEEC and ECC-1 whilst, the lipid-raft protein FLOT1 demonstrated punctate staining in the apical surface of ECC-1 plasma membranes and was upregulated in the epithelium in the receptive phase of the menstrual cycle (p lower or equal 0.05).

**Conclusions:**

This is the first study to use a proteomics approach to identify hEEC plasma membrane proteins that may be useful as infertility markers or pharmacological targets for fertility regulation.

## Background

Embryo implantation is a complex process requiring synchronised endometrial receptivity and blastocyst competence [1]. The initial apposition, attachment and adhesion of the blastocyst to an adequately prepared or receptive maternal endometrium occurs via a co-ordinated dialogue of locally produced molecules, including cytokines, adhesion and extracellular matrix (ECM) molecules [2].

Interleukin (IL) 11 belongs to the IL6 family of cytokines and signals via a heterodimeric complex of IL11 receptor (R) α and gp130 [3]. IL11 is absolutely required for decidualization of endometrial stromal cells and blastocyst implantation in mice [4-6]. Female mice with a null mutation of the IL11-Rα are infertile due to a defective post-implantation response to the implanting blastocyst. There is evidence supporting the importance of IL11 in the initial attachment of the human blastocyst to the endometrium. While IL11 is produced only by decidual cells post-implantation in mice, in humans IL11 is also expressed by endometrial glandular and luminal epithelium in the mid-secretory (receptive) phase of the menstrual cycle when blastocyst implantation is most likely to occur [7-10]. Similarly, mid-secretory phase luminal and glandular epithelium express IL11-Rα [7,8,11,12]. Both epithelial IL11 and IL11-Rα are dysregulated in the endometrium of infertile women compared with that of fertile controls [13]. There is a positive correlation between IL11 levels in uterine flushings (collected during the window of endometrial receptivity) and the responsiveness of women to ovarian stimulation prior to IVF [14]. This suggests that reduced IL11 secretion by endometrial epithelial cells during this time may be responsible for the reduced implantation/pregnancy rates in excessive ovarian responders during IVF treatment.

We have previously demonstrated that IL11 regulates epithelial cell adhesive properties suggesting that IL11 regulates cell surface proteins to facilitate the adhesion of the blastocyst to the endometrial epithelium [15]. The downstream mechanisms by which IL11 regulates endometrial epithelial cell adhesion are poorly understood.

The aims of the present study were to compare the protein profiles of IL11 treated and untreated human endometrial epithelial cell membranes by 2D-DIGE and identify the differentially expressed proteins by mass spectrometry (MS). The second aim was to validate some of the differentially regulated proteins by Western immunoblotting and to determine their cellular location in human endometrium by immunohistochemistry. This is the first study to use a proteomic approach to define the mechanisms by which IL11 regulates endometrial plasma membrane proteins. This study has identified two proteins, which are known regulators of cell adhesion and are likely to be critical for endometrial epithelial cell adhesion, a process that is absolutely required for embryo implantation.

## Methods

### Patients and tissues

Endometrial tissues were obtained at curettage from women with normal menstrual cycle (ranging from d28-32) and no apparent endometrial dysfunction that were scheduled for tubal ligation or were undergoing testing for tubal patency. The stage of the cycle was determined from the patient's testimony and confirmed histologically by a qualified gynecological pathologist. The age range of the women was 32-40 years. For immunohistochemistry studies: endometrial samples were collected across different stages of the menstrual cycle *(n = 6-8/cycle); *menstrual (d1-4), proliferative (d5-14), early secretory (d15-19), mid-secretory (d20-24) and late secretory (d25-32) phases. Written informed consent was obtained from each patient and the study was approved by Southern Health Human Research and Ethics committee. All endometrial biopsies were fixed overnight in 4% neutral buffered formalin, prior to routine paraffin embedding.

### Cell culture

Human endometrial tissues were digested with collagenase (n = 6), and the suspension was filtered through 43 and 11 μm nylon mesh to collect endometrial epithelial glands, as previously described work [15]. The cells/epithelial fragments were collected and re-suspended in a 1:1 mixture of DMEM/Hams F-12 (F12; Thermo Electron Corp., Melbourne, Australia) supplemented with 10% fetal calf serum (FCS; Invitrogen, Carlsbad, CA), 2 mM L-glutamine (Thermo Electron), and 1% antibiotic-antimycotic solution (Life Technologies, Inc., Auckland, New Zealand) and plated. Endometrial epithelial cells (hEEC) were collected from the filter paper and further purified through selective adherence. Briefly, epithelial glands were serially repeated (three times) in plastic culture dishes for 30 min to allow adherence of contaminating stromal cells. Non-adherent cells/glands were transferred to 24-well plates and epithelial cells were allowed to grow out from glandular structures for 48 h. A purity of greater than 95% was necessary for the cells to be used experimentally. Confluent hEEC were cultured in serum-free 1:1 DMEM/F12, 2 mM L-glutamine, and 1% antibiotic-antimycotic solution. Confluent cells of 4-6 wells of 24 well plates of pure primary hEEC were used as starting material (approximately 0.2 million cells).

The endometrial epithelial carcinoma cell line; ECC-1 and Ishikawa cells were cultured in DMEM/F12 (1:1) and DMEM respectively (Invitrogen, Victoria, Australia) supplemented with 10% fetal calf serum (SAFC Biosciences, Victoria, Australia), 1% L-glutamine (Sigma-Aldrich Pty. Ltd, Sydney, Australia) and 1% antibiotic-antimycotic (Invitrogen, Victoria, Australia). Confluent cells (1 × T175 cm^2 ^flask, starting material of approximately 15 million cells) were transferred into their respective serum reduced (1% FCS) medium for 24 h prior to treatment. For proteomic analyses, only ECC-1 cells were used but an additional cell line; Ishikawa cells was also included in the proteomic validation. There were two different treatment groups (n = 4 per group) of control (media alone) and IL11 (100 ng/ml, concentration of IL11 determined from previous studies in endometrial cells) [15]. An additional group of IL11 (500 ng/ml) was included for validation of proteomic analyses by Western blot. For Western blot validation, 1 × T75 cm^2 ^flask of confluent cells per treatment was used as starting material (approximately 5-6 million cells) (n = 3 in duplicate per treatment group).

### Protein extraction and membrane purification

Prior to protein extraction, cells were washed ten times with phosphate buffered saline free of Ca^2+ ^and Mg^2+ ^(PBS^-^) and collected in Tris buffer (pH 7.4) containing protease inhibitor cocktail set (Calbiochem, Darmstadt, Germany). All subsequent steps were performed on ice.

#### Cellular membrane protein extraction

Briefly, the cell lysates were homogenised by repeated passaging through a syringe and needle, using both 21 G and 25 G needles. This was followed by centrifugation at 800 g for 15 min at 4°C, after which the supernatant was transferred to a fresh tube and the pellet re-extracted as above. The two supernatants were combined and centrifuged using the Optima™ TXN ultracentrifuge (Beckman Coulter, Inc., USA) at 100,00 g for 45 min at 4°C. The supernatant containing soluble cytosolic proteins was transferred to a fresh tube and snap frozen at -80°C. An aliquot (cytosol (C)-fraction) was collected for later analysis by Western blot for purity. The pellet was re-suspended with Tris buffer and centrifugation repeated as above. The supernatant was discarded and the protein pellet containing crude membrane proteins was re-suspended in solubilisation buffer (40 mM Tris, 7 M urea, 2 M thiourea, 1% (w/v) C_7_BzO (3-(4-Heptyl)phenyl-2-hydroxypropyl) dimethylammoniopropanesulfonte). An aliquot (membrane (M)-fraction) was collected for analysis by Western blot for purity.

#### Total cellular protein extraction

Briefly, the cell lysates were lysed and scraped in the buffer mentioned above. Cell extracts were then centrifuged at 280 g for 30 min at 4°C and supernatant collected for analysis by Western blot.

### Protein concentration measurement

The protein concentrations of membrane samples were determined using Bradford Assay kit (Bio-Rad, Hercules, CA) according to manufacturer's instructions. Subsequently, membrane proteins were precipitated with acetone for 90 min, centrifuged at 10,000 g for 20 min at room temperature. The protein pellet was air dried and then dissolved in labeling buffer (30 mM Tris/HCL, pH 8.1, 7 M urea, 2 M thiourea, 1% (w/v) C_7_BzO). The final protein concentration was adjusted to 5 μg/μl with labeling buffer, snap frozen on dry ice and stored at -80°C until use.

### Western blot analysis

To investigate the purity of membrane isolation, Western blot analyses were performed. Both Membrane (M) and Cytosol (C) fraction extracts were resolved on 11% SDS-PAGE gels and transferred to nitrocellulose membranes. The same amount of protein (30 μg) was loaded. All membranes were incubated with Ponceau-S (Sigma) to ensure equal protein loading in all lanes. The membranes were blocked with 5% nonfat dry milk in TBS with 0.1% Tween 20 (Bio-Rad Laboratories, Hercules, CA). Each membrane was then incubated with one of the following primary antibodies (at a dilution of 1:500 in 5% skim milk/TBS) at 4°C overnight. Primary antibodies included mouse monoclonal anti-Na/K-ATPase, rabbit polyclonal anti-GRP94 (glucose-regulated protein 94 kDa), mouse monoclonal anti-Ku-86 and mouse monoclonal anti-GAPDH (all were from Santa Cruz Biotechnology Inc.; Santa Cruz, CA) for detecting plasma membrane, endoplasmic reticulum, nuclear and cytoplasm markers, respectively. After washing with 0.2% Tween-20/TBS, the membranes were further incubated with respective secondary antibodies conjugated with horseradish peroxidase (1:2000 in 5% skim milk/TBS) (Dako Cytomation Glostrup, Denmark) at room temperature for 1 h. HRP activity was detected using enhanced chemiluminescence reagent (Pierce, Rockford, IL, USA).

### 2D-DIGE labeling of membrane proteins

Fluorescent labeling of proteins was performed using minimal CyDye labeling methodology as described by the manufacturer (GE Healthcare Bioscience, Uppsala, Sweden) and detailed elsewhere [16,17]. Briefly, labeling was carried out on ice in the dark using 200 pmol dye per 50 μg protein at a protein concentration of 5 μg/μl for 30 min. The labeling reaction was quenched by the addition of 1/10 vol of 10 mM lysine. Samples were either labeled with Cy3 or Cy5 with an internal standard (comprised of equal amounts of protein from each sample) labeled with Cy2. On completion of labeling, the samples were combined as recommended by the manufacturer. The combined samples were then precipitated in acetone as described above and the dried protein pellet re-suspended in 450 μl of the solubilisation buffer (40 mM Tris, 7 M urea, 2 M thiourea, 1% (w.v) C_7_BzO) prior to isoelectric focusing.

Isoelectric focusing (IEF) was performed using 24 cm immobilized pH gradient strips (IPGs) covering the pH range of 3-10, Tracking Dye, DTT to 100 mM and ampholytes to 0.5% (v/v) were added and each sample loaded by passive rehydration for 6 h at RT [17]. IPG strips were focused overnight using an IPGphor instrument (GE Healthcare, BUCKS, UK), according to the following profile: current limited to 60 μA per strip, 100 V for 90 min, 300 V for 90 min, 500 V for 3 h, gradient to 1000 V for 4 h, gradient to 8000 V for 3 h and then constant 8000 V overnight until reaching between 60 000 and 80 000 Vh. Following focusing, the strips were incubated in 10 ml of equilibration buffer (50 mM Tris/HCL, pH 8.8, 6 M urea, 30% (v/v) glycerol, 2% (w/v) SDS, 0.01% (w/v) bromophenol blue) for 20 min on a shaking platform at 80 rpm. Second dimension PAGE was performed on 8-16% gradient polyacrylamide gels (24 × 24 cm) overnight in a BioRad Dodeca cell (Richmond, CA) at 50 V as described by the manufacturer. The run was terminated when the dye front had reached the bottom of the gel.

2D gels were scanned using a Fuji FLA5100 laser scanner (Fujifilm, Tokyo, Japan) at excitation wavelengths of 473, 535 and 635 nm and with specialty dual wavelength emission filters for Cy2, Cy3 and Cy5 respectively. Image alignment, spot detection, background removal and expression analysis were performed using PG240 SameSpots software (Nonlinear Dynamics, Newcastle Upon Tyne, UK).

### Protein identification by LCMS/MS mass spectrometry

Spots of interest were excised from the gel using a ProPicII robotic spot picker (Genomic Solutions, MI) based on X-Y coordinates exported directly from PG240 SameSpots. Protein identification by MS/MS was carried out as described [17]. Monoisotopic peak masses were automatically extracted using GPS Explorer software (v3.0 build 311; Applied Biosystems, CA) and peak lists searched against non-redundant UniProtKB/Swiss-Prot database (release 56.2; 20407 human sequence entries) [18] using the MASCOT search engine (updated 03/01/2007) [19]. Species was restricted to *Homo sapiens*, carbonylamide-cysteine (CAM, fixed modification) and oxidation of methionine (variable modification) were taken into account, a parent ion mass tolerance of 60 ppm and 1 missed cleavage (trypsin) was allowed. Up to fifteen of the most intense peptides detected in each MS scan were automatically selected for MS/MS analysis. Peak lists were extracted using Data Analysis software version 3.4 (Bruker Diagnostics, Germany). The parameters used to create the peak list (version 1.1, Matrix Science) were a mass range of 700-5000 Da and signal-to-noise threshold of 5, against the UniProtKB/Swiss-Prot database as above, with fragment mass tolerance of 0.1 Da. Protein identities were assigned using the following criteria to evaluate the search; MOWSE score, number and intensity of peptides matched, and direct correlation between the identified protein, its estimated molecular mass and pI determined from the 2D gel (see Additional file 1, Table S1).

### Validation of proteins by Western blotting

Total lysates and membrane proteins extracted from control and IL11 treated cells for both endometrial epithelial cell lines (ECC-1 and Ishikawa) were resolved on 11% SDS-PAGE gels and transferred to nitrocellulose membranes. 0.1 μg and 10 μg of membrane protein were used for validation of ANXA2 and FLOT1 respectively. However, higher membrane protein load was needed for validation in the primary EEC. Furthermore, all membranes were incubated with Ponceau-S (Sigma) to ensure equal protein loading in all lanes. Non-specific bindings were blocked with 5% nonfat dry milk in TBS with 0.1% Tween 20 (Bio-Rad Laboratories, Hercules, CA) and probed separately with antibodies specific for ANXA2 (Abcam, UK, # 41803) and FLOT1 (Cell Signalling Inc., US, #3253) at 4°C overnight. The membranes were washed in TBS with 0.2% Tween 20 and incubated for 1 h with horseradish peroxidase (HRP)-conjugated rabbit secondary antibody (1:2000; Dako Cytomation Glostrup, Denmark). HRP activity was detected using enhanced chemiluminescence reagent (Pierce, Rockford, IL, USA). All *in vitro *cell culture experiments were carried out in 3 independent experiments.

### Validation of proteins by immunohistochemistry

#### Formalin-fixed human endometrial biopsies

Localisation of FLOT1 across the menstrual cycle was examined in endometrial tissue sections from different phases of the cycle (menstrual, n = 7; proliferative, n = 8; early secretory, n = 8; mid-secretory, n = 7, late secretory, n = 9) using the same antibody as employed for Western blotting. In brief, paraffin sections (5 μm) were dewaxed in histosol, dehydrated in a graded series of ethanol and then microwaved at high power (700 W) in 0.01 M citric acid buffer (pH 6.0) for 5 min. Endogenous peroxidase activity was quenched with 6%H_2_O_2 _in 100% methanol (1:1 v/v) for 10 min in the dark at room temperature. Non-specific binding was blocked by incubation with non-immune blocking solution of 10% normal goat serum and 2% normal human serum, diluted in 1 × Tris-buffered saline (TBS) for 30 min. Primary antibody was diluted 1:100 (Cell Signalling Inc., US, #3253) in blocking solution and applied overnight at 4°C. Antibody localisation was detected by sequential application of biotinylated goat anti-rabbit IgG diluted 1:200 in blocking solution for 30 min and an avidin-biotin complex conjugated to HRP (Vectastain ABC Elite kite; Vector Laboratories, Burlingame, CA, USA). The substrate used was diaminobenzidine (DAB) (Zymed, San Francisco, USA) forming an insoluble brown precipitate. Sections were counterstained with Harris hematoxylin (Sigma Chemicals, St. Louis, MO), dehydrated and mounted with DPX. Negative isotype controls were included for each section where non-immunised rabbit IgG was substituted at matching concentration to the primary antibody. Positive control of human tonsil was also included in the run.

The relative intensity of the immunostaining was scored semi-quantitatively by two independent observers on a scale of 0 (no staining) to 3 (maximal staining) in each of the cellular compartments.

#### Primary endometrial epithelial cells (hEEC) and cell line; ECC-1 immunocytochemistry

Both primary hEEC and ECC-1 cells were cultured in control (media only) and IL11 (100 ng/ml) for 24 hr. Cells were trypsanised and cell suspension collected and centrifuged at 438 g for 10 min. The supernatant was discarded and cell pellet re-suspended in 1 ml of PBS^-^. 200 μl (containing 300,000 cells/ml) was applied per well (4 well chamber) by cytocentrifugation at 4000 U/min (Hettich Centrifuge, Universal 16A) for 10 min. Slides were air-dried overnight at room temperature, then fixed in 70% ethanol for 10 min.

Immunocytochemistry was carried out as above. Briefly, primary antibody was diluted 1:2000 and 1:1000 (ANXA, Abcam, UK, # 41803) and 1:20 and 1:50 (FLOT1, Cell Signalling Inc., US, #3253) respectively for primary hEEC and ECC-1 cells in blocking solution and applied overnight at 4°C. Cytospins were used instead of chamber slides for immunohistochemistry to validate as we have previously trialled seeding the monolayer cells onto glass chamber slides that are suitable for immunohistochemistry but the cells did not adhere well.

### Statistical analysis

Fold changes and all statistical analyses for proteomic analyses were calculated based on normalised spot volumes where the internal standard was used to perform normalisation using PG240 SameSpots software. Statistical analysis of immunoblotting and immunohistochemistry results was carried out using GraphPad Prism (GraphPad Software Inc., CA), with all data expressed as mean ± SEM. Statistical analysis of endometrial immunostaining intensity was performed using one-way ANOVA with Bonferroni's post-test, while for Western blot analysis student's *t-test *was used. A *p *value ≤ 0.05 was considered statistically significant.

## Results

**Figure 1:**
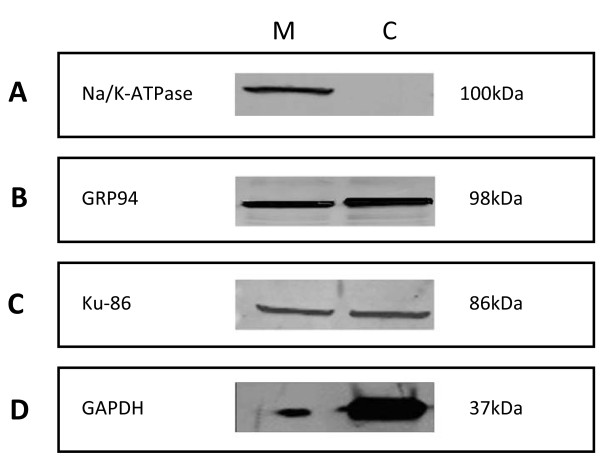
**Evaluation of the purity of the cell membrane isolation method**. Cells were subjected to crude isolation of Membrane (M) and Cytosol (C) fractions. Western blot analysis of the different markers - (A) Na/K-ATPase (plasma membrane), (B) GRP94 (endoplasmic reticulum), (C) Ku86 (nuclear) and (D) GAPDH (cytosol). (M) Membrane, (C) Cytosol and (Mw) molecular weight marker (kDa).

The membrane and cytosol fractions were isolated from the human endometrial epithelial cell (hEEC) line, ECC-1, using several centrifugation steps. To test for cell membrane protein purity, we compared the two fractions obtained in our isolation method (Membrane [M] and Cytosol [C]) using different organelle markers. An equal amount of protein for the M and C fractions was subjected to Western blot analysis (Figure 1) to examine the abundance of (A) Na/K-ATPase, (B) GRP94, (C) Ku-86 and (D) GAPDH which are plasma membrane, endoplasmic reticulum, nuclear and cytoplasm markers respectively. Na/K-ATPase protein was observed only in the M fraction, indicating a successful enrichment of plasma membrane proteins in the M fraction. In contrast to Na/K-ATPase, GRP94 and Ku-86 were present in both fractions (M and C fractions) but GRP94 protein abundance was slightly higher in the C fraction compared to the M fraction, while the abundance of Ku-86 was equal between the M and C fractions. GAPDH protein was maximal in the C fraction. Ponceau-S was used to examine the level of protein for each sample transferred to the nitrocellulose membrane during Western blot analysis (data not shown) in addition to loading of equal amounts of proteins.

### Characterisation of proteomic profiles on the membrane surface of ECC-1

**Figure 2:**
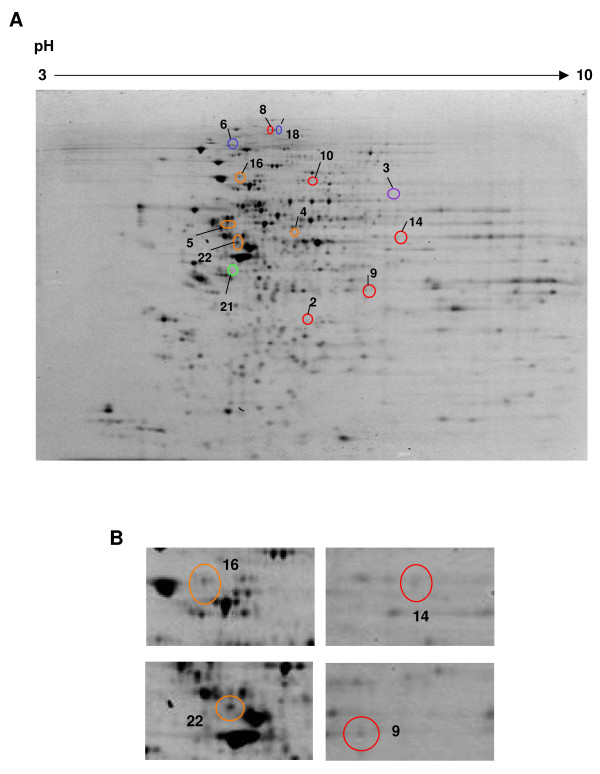
**Representative 2D SDS-PAGE image of proteins from the human endometrial carcinoma epithelial cell line (ECC-1)**. Proteins (150 μg) extracted from membrane fraction of ECC-1 cells were separated by 2D PAGE (first dimension pH 3-10, second dimension 8-16% polyacrylamide gradient). (A) A representative gel of the pooled internal standard (Cy2) is shown. Overall 866 spots were resolved. Proteins were clustered according to similarities in their expression patterns (grouped by colours). (B) Differentially expressed proteins were identified and spots of interest were chosen for further analysis by LCMS/MS are numbered and indicated by circles.

The abundance of membrane proteins in ECC-1 cells treated with IL11 or diluent control was assessed by 2D DIGE (analytical replicates of 4 independent experiments). A total of 866 spots were resolved by the 2D DIGE analysis following treatment with IL11 (Figure 2A). Protein spots displaying statistically significant changes between the control and IL11 treatment groups (p ≤ 0.05) were considered to be present at altered levels. Thirteen spots were significantly altered between the IL11 treated and diluent control-treated ECC-1 cell membranes (Figure 2A). Four protein spots (Figure 2B) that were altered between IL11 and control-treated cells were chosen for identification by mass spectrometry based on the following criteria: a fold change of ≥ 1.3 and a spot volume of ≥ 8000, both cross-referenced with the ability to visualise the spot on the gel. Twenty unique proteins were identified by MS analysis and are summarised in Table 1. Each spot analysed by mass spectrometry contained a mixture of proteins.

**Table 1:** Assigned Protein Identification Using LC-MS/MS

Spot	p-value	Fold-change	Protein ID	ACC. No. (Swissprot)	Gene	Sequence coverage (%)	No. peptides identified	Mr/pI
9*	0.02	1.33	Annexin A2	P07355	*ANXA2*	38.6	13	38/7.56
			Ribose-phosphate pyrophosphokinase 1	P60891	*PRPS1*	15.1	4	35/6.56
			Voltage-dependent anion-selective channel protein 2	P45880	*VDAC2*	17.70	5	32/7.5
			Dual specificity mitogen-activated protein kinase kinase 6	P52564	*MAP2K6*	11.4	3	37/7.01
14*	0.04	1.40	Flotillin-1	O75955	*FLOT1*	62.3	23	47/7.08
			Alpha-enolase	P06733	*ENO1*	6.68	2	47/6.99
16*	0.04	-1.32	Cdc42-interacting protein 4	Q15462	*TRIP10*	50.6	22	69/5.55
			78 kDa glucose-regulated protein (GRP78)	P11021	*HSPA5*	47.6	32	72/5.01
			Collagen type IV alpha-3 binding protein	Q9Y5P4	*COL4A3BP*	20.7	9	71/5.29
			NADH-ubiquinone oxireductase 75 kDa subunit, mitochondrial	P28331	*NDUFS1*	13.2	8	79/5.42
			Stress-70 protein, mitochondrial	P38646	*HSPA9*	11.5	7	74/5.44
22*	0.04	-1.32	cDNA FLJ5299	A8K781	*cDNA FLJ5299*	47.0	17	47/5.11
			Keratin, type II cytoskeletal 8	P05787	*KRT8*	46.8	25	48/5.34
			Thioredoxin domain-containing protein 5	Q8NBS9	*TXNDC5*	27.3	11	48/5.37
			Actin, cytoplasmc 1	P60709	*ACTB*	26.4	9	42/5.29
			26S protease regulatory subunit 6B	P43686	*PSMC4*	22.7	9	47/5.09
			Uncharacterised protein KIAA0174 (ISTI homolog)	P53990	*KIAA0174*	14.6	5	40/5.22
			Eukaryotic initiation factor 4A-1	P60842	*EIF4A1*	16.0	5	46/5.32
			Hsc70-interacting protein	P50502	*ST13*	12.5	4	41/5.18
			Heterogenous nuclear ribonucleoprotein F	P52597	*HNRNPF*	19.3	4	46/5.38

^a ^Asterisk (*) denotes spots identified as containing a mixture of 2 or more proteins, or where protein isoforms cannot be distinguished based on peptides identified.

P-value and fold-change obtained based on normalised spot volume between control and IL11 treated group.

### Validation of membrane protein abundance changes between IL11 treated and control treated hEEC cells

Protein changes observed between IL11 and control treated cells were validated by Western blotting and immunocytochemistry. Proteins were validated by western blot in two hEEC cell lines: ECC-1 and Ishikawa cells whilst, immunocytochemistry was carried out in ECC-1 cells and primary hEEC. Endometrial biopsies were also used to assess the cellular localisation and relative level of protein production *in vivo*. Immunohistochemical validation was performed using endometrial tissues from women with normal fertility throughout the menstrual cycle (*n = 6-8/cycle)*.

#### IL11 increased Annexin A2 and Flotillin-1 proteins in hEEC membranes

**Figure 3:**
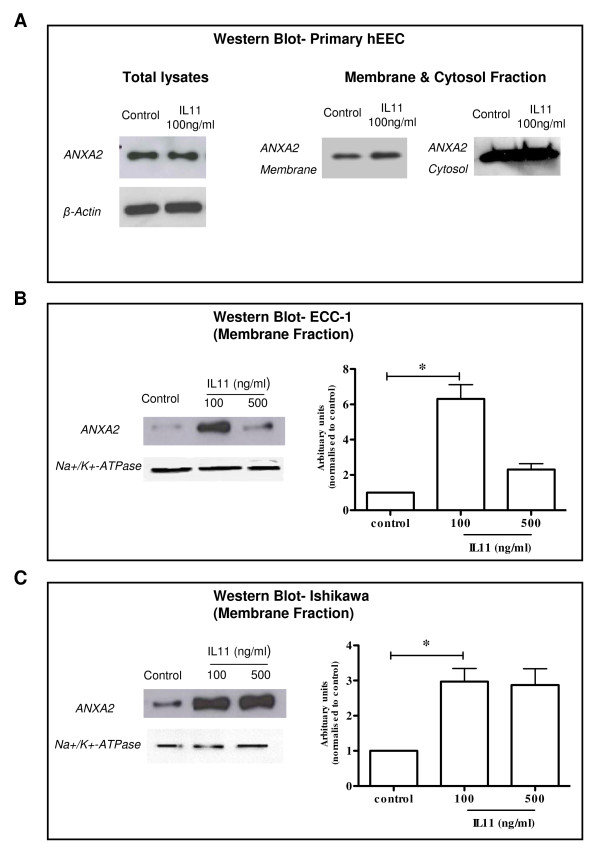
**Western blot analysis of IL11 regulated Annexin A2 (ANXA2) protein in human endometrial epithelial cell (hEEC) membranes and in total cellular protein**. The hEEC cell lines, ECC-1 and Ishikawa and primary hEEC cells were treated without (diluent control) or with IL11 (100 and/or 500 ng/ml) for 24 h and membrane proteins extracted as described in the Methods. Cell lysates were electrophoresed by SDS-PAGE and immunoblotted with ANXA2 in (A) total primary hEEC protein lysates **(**30 μg; left panel) and membrane primary hEEC (1 μg; right panel), (B) ECC-1 (0.1 μg) and (C) Ishikawa (0.1 μg) cells. Na^+^/K^+^-ATPase is used as a loading control for membrane proteins. Data is shown as a single photomicrograph representative of 3 independent experiments. * *P *≤ 0.05; *t-test*.

DIGE analysis identified that annexin A2 (ANXA2) in protein spot 9 (Table 1) was significantly increased following IL11 treatment. A mixture of four proteins were identified in spot 9 and ANXA2 was chosen due to the highest number of peptides present in the analyses and our interest in its biological function. Western blot analysis revealed that ANXA2 was present as a single band of molecular weight ~37 kDa (Figure 3A-C) and was significantly increased in the membrane protein fraction in IL11 treated compared to control in both the primary hEEC (Figure 3A, right panel) and hEEC-derived cell lines; ECC-1 (Figure 3B, left panel) and Ishikawa (Figure 3C, left panel) but no differences were observed in the total protein fractions of IL11 treated compared to control treated primary hEEC (Figure 3A; left panel). However, there appear to be a difference in abundance in IL11 treated membrane fraction but no difference in the cytosol fraction in both the control or IL11 treated (Figure 3A; right panel). In addition, Western blot showed that ANXA2 abundance was upregulated 2-fold and 2.5-fold in IL11 treated compared to control in the cell membrane fraction in both ECC-1 (100 ng/ml, p ≤ 0.05) (Figure 3B; right panel) and Ishikawa cells (100 ng/ml, p ≤ 0.05) (Figure 3C; right panel) respectively. Na^+^/K^+^-ATPase was used as an internal loading control in addition to Ponceau-S staining to visualise equal loading (data not shown). Equal concentrations of total membrane protein was loaded per fraction as detailed in the methods section.

**Figure 4:**
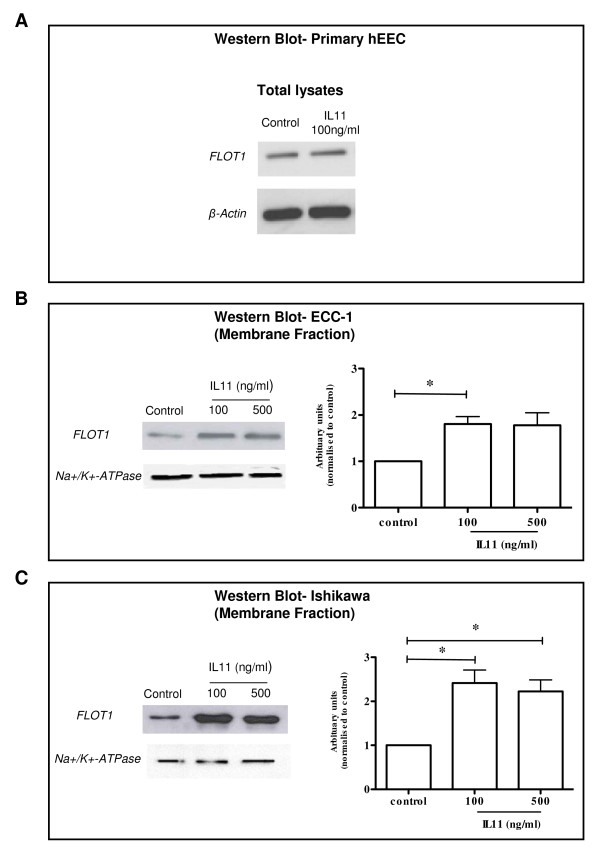
**Effect of IL11 on flotillin-1 (FLOT1) in human endometrial epithelial cell (hEEC) membranes and in total cellular protein**. The hEEC cell lines, ECC-1 and Ishikawa and primary hEEC cells were treated without (diluent control) or with IL11 (100 and/or 500 ng/ml) for 24 h and membrane proteins extracted as described in the Methods. Cell lysates were electrophoresed by SDS-PAGE and immunoblotted with FLOT1 in (A) total primary hEEC protein lysates **(**30 μg), (B) ECC-1 (10 μg) and (C) Ishikawa (10 μg) cells. Na^+^/K^+^-ATPase is used as a loading control for membrane proteins. Data is shown as a single photomicrograph representative of 3 independent experiments. * *P *≤ 0.05; *t-test*.

Flotillin-1 (FLOT1) (spot 14; Table 1) was also identified to be significantly increased following IL11 treatment. Western blot revealed that FLOT1 was present as a single band of molecular weight ~49 kDa (Figure 4A-C). There was no difference in FLOT1 total protein in the IL11 treated compared to control treated primary hEEC (Figure 4A). However, quantitative analysis demonstrated a 1.8-fold (100 ng/ml; p ≤ 0.05) and 2.3/2.4-fold (100 and 500 ng/ml; p ≤ 0.05) increase in total FLOT1 protein abundance on the cell surface membranes of ECC-1 (Figure 4B; right panel) and Ishikawa cells respectively (Figure 4C; right panel). 78 kDa glucose-regulated protein (GRP78) was also analysed by Western blot of primary hEEC membranes, however, following IL11 treatment there was no statistically significant decrease in abundance compared to control treated cells (data not shown).

#### Immunolocalisation of ANXA2 and FLOT1 in hEEC cells and human endometrial tissue

**Figure 5:**
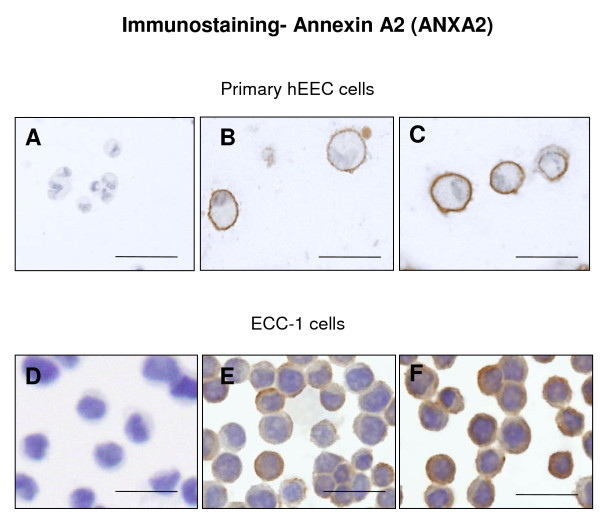
**Photomicrograph representing immunocytochemical staining for annexin A2 (ANXA2) in human endometrial epithelial cells (hEEC)**. Primary hEEC and the cell lines ECC-1 were treated with diluent or IL11 (100 ng/ml) for 24 h. Cytospins were prepared and subjected to immunocytochemistry as described in the Materials & Methods. Positive staining for ANXA2 showed as brown pigment with blue nuclear counterstain. (A, D) Negative IgG (B) Control primary EEC (C) IL11 100 ng/ml primary hEEC (E) Control ECC-1 (F) IL11 100 ng/ml ECC-1. Bars represent 200 μm.

Immunolocalisation for ANXA2 in human endometrial epithelium during the secretory phase of the menstrual cycle has previously been demonstrated [20]. IL11 treatment induced stronger ANXA2 expression on the cell surface of primary hEEC (Figure 5B-C) and ECC-1 (Figure 5E-F) cells *in vitro*. Negative isotype IgG control tissues did not stain positively (Figure 5A, D).

**Figure 6:**
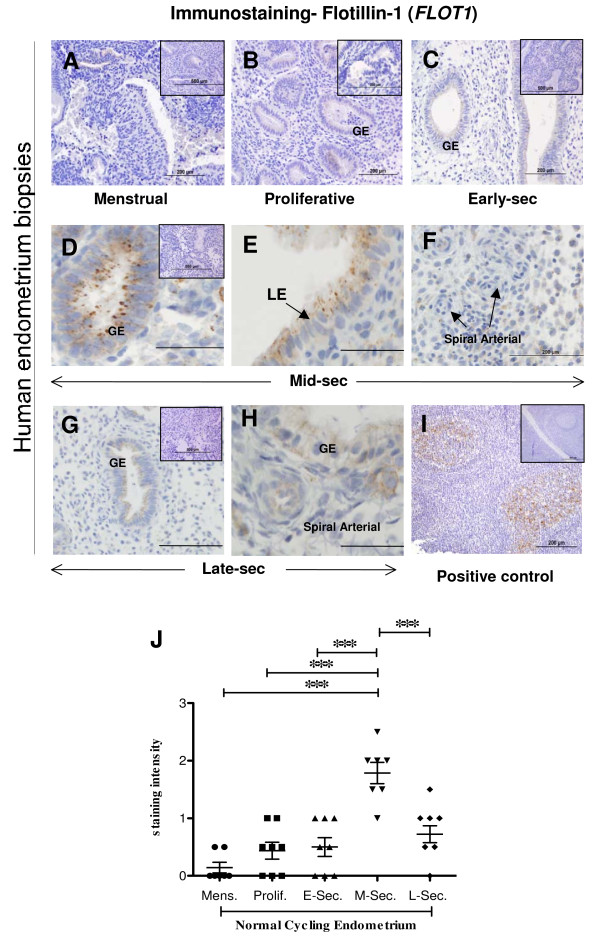
**Photomicrograph representing immunohistochemical staining for flotillin-1 (FLOT1) in human endometrial tissue throughout the menstrual cycle and in ECC-1 cells**. Positive staining for FLOT1 showed as brown pigment with blue nuclear counterstain. (A) Menstrual, d1-4 (B) Proliferative, d5-14 (C) Early-secretory, d15-20 (D-F) Mid-secretory, d21-24 (G-H) Late-secretory, d25-d27. (GE) Glandular epithelial, (LE) Luminal epithelial. Insets in each case are matched negative controls. Positive control of human tonsil included (I). Bars represent 200 μm. Semi-quantitative analysis of staining intensity for FLOT1 in GE (J). Data represented as mean ± SEM. * *P *≤ 0.05, ** *P *≤ 0.01, *** *P *≤ 0.001, *Kruskal Wallis test, with Dunn's post-hoc test*.

Localisation of FLOT1 in human endometrium throughout the menstrual cycle revealed that FLOT1 was restricted to the glandular epithelial cells (Figure 6A-D and 6G). Maximal staining for FLOT1 was observed in the mid-secretory phase endometrium and was significantly more intense (p ≤ 0.0001) in the glands of the mid-secretory phase endometrium compared to all other phases (Figure 6J). In the endometrial epithelial cells, strong punctate staining for FLOT1 was present in the apical surface of the plasma membranes, while less intense staining was present basolaterally (Figure 6D). Intense punctuate staining was also observed in the apical cell surface in luminal epithelial cells only in the mid-secretory phase of the cycle (Figure 6E). Luminal epithelial cells from all other phases of the menstrual cycle did not show positive staining for FLOT1 (data not shown). Mild staining for FLOT1 was observed in the vascular endothelial and smooth muscle cells in mid and late secretory phase endometrium (Figure 6F and 6H). Semi-quantitative analysis revealed that there is a significant increase of FLOT1 expression in the mid-secretory phase of the endometrium (Figure 6J). A positive control of human tonsil was also included and demonstrated positive staining (Figure 6I). Negative isotype IgG control tissues shown as insets in Figure 6A-D,G.

**Figure 7:**
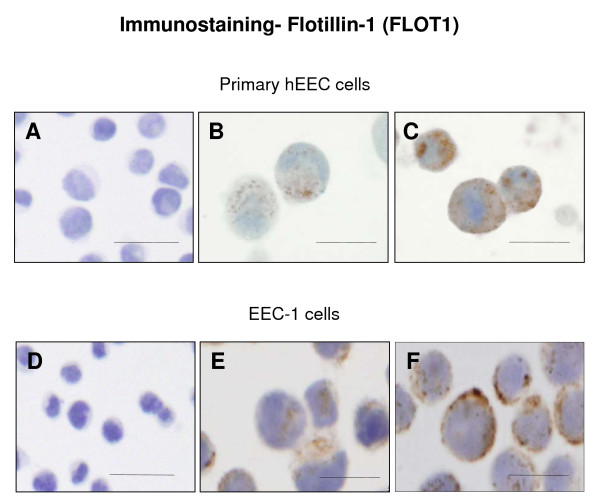
**Photomicrograph representing immunocytochemical staining for flotillin-1 (FLOT1) in human endometrial epithelial cells (hEEC)**. Primary hEEC and the cell lines ECC-1 were treated with diluent or IL11 (100 ng/ml) for 24 h. Cytospins were prepared and subjected to immunocytochemistry as described in the Materials & Methods. Positive staining for FLOT1 showed as brown pigment with blue nuclear counterstain. (A, D) Negative IgG (B) Control primary EEC (C) IL11 100 ng/ml primary hEEC (E) Control ECC-1 (F) IL11 100 ng/ml ECC-1. Bars represent 200 μm.

We further examined FLOT1 localisation in both primary hEEC and ECC-1 cultured cells. FLOT1 localised to both the membrane and cytosolic (Figure 7A-D) component of the cells but it was enhanced following IL11 treatment, with stronger punctate staining in primary hEEC isolated from the proliferative stage of the menstrual cycle (Figure 7B) and membrane staining in ECC-1 cells (Figure 7F). There was no effect in hEEC isolated during the secretory stage (data not shown).

## Discussion

Several studies have previously sought to define the importance of IL11 in facilitating embryo implantation in mice and in humans [4,9,15]. *In vitro*, IL11 regulates hEEC adhesive properties however, to date no proteins have been identified to act downstream of IL11 on the membrane surface that may facilitate cell adhesion. In this study, we identified two candidate proteins (ANXA2 and FLOT1) upregulated by IL11 in the endometrial epithelial cell membrane using a 2D-DIGE approach. Here, we found that IL11 up-regulated membrane ANXA2 in both endometrial epithelial cell lines and also primary hEEC. Interestingly, assessment of total protein in primary hEEC showed no differences in ANXA2 expression, suggesting that IL11 did not regulate total ANXA2, highlighting the importance of pre-fractionating cellular proteins prior to proteomic analysis.

This study also identified that IL11 treatment of ECC1 cells down-regulated a number of spots with a maximal fold decrease of 1.32. We validated one identified down-regulated protein, GRP78, in ECC1 and primary hEEC by Western blot. While we did show a slight decrease in GRP78 protein abundance following IL11 treatment compared to control, this did not reach statistical significance. This may be due to the fold change representing the overall fold change of the spot rather than of the individual proteins. We did however, validate the upregulated protein ANXA2: Annexin A2 is a calcium-dependent phospholipid binding protein [21] and is present in intracellular, membrane and secreted forms [21,22]. ANXA2 interacts with cell matrix and proteases to regulate cell migration and adhesion - for instance it can act as a receptor for secreted serine proteases on endothelial cells [23,24]. In a study investigating the endometrium of women using IUDs for contraceptive purposes, ANXA2 was shown to be up-regulated in the receptive compared to the pre-receptive endometrium [20]. Numerous studies show ANXA2 is involved in cell adhesion and actin cytoskeletal rearrangements [23,25,26] as well as increasing cell adhesion molecule production [27]. Therefore, we suggest that ANXA2 expression on the epithelial membrane surface predisposes the epithelium to being receptive towards blastocyst attachment during embryo implantation.

FLOT1 immunolocalisation to the endometrial glandular and luminal epithelium was maximal during the receptive phase of the menstrual cycle, correlating with the period of maximal IL11 immunolocalisation [7-10]. Up-regulation of membrane bound FLOT1 upon IL11 stimulus was also found here in both cell lines. Conversely, localisation of FLOT1 in cultured primary hEEC was maximal upon IL11 stimulus in hEEC isolated during the proliferative stage but there was no difference in hEEC isolated during the secretory stage. We suggest this is due to the high levels of FLOT1 found in the epithelium during the secretory stage, thus, it was not possible to detect an increase in FLOT1 expression, whereas, the absence of FLOT1 in the proliferative stage epithelium allowed detection of FLOT1 stimulated following IL11 treatment. Further, we suggest we were unable to detect FLOT1 in primary hEEC by Western blot because of the unfeasibly large number of cells required to detect a low abundance protein (as demonstrated by cytospin immunolocalisation) such as this.

FLOT1 is a lipid raft associated membrane protein and is endocytosed from the plasma membrane into intracellular compartments [28]. FLOT1 has also been shown to reside in the plasma membrane, as well as in the cytoplasm [29] as observed in our cytospin data. Recent findings demonstrate that FLOT1 is associated with cell motility and transformation, membrane trafficking, phagocytosis and epidermal growth factor receptor signaling [30]. Given that FLOT1 localises to the cell membrane lipid rafts, it is not surprising that it is also involved in cellular communication, such as cell-cell contacts, focal adhesions, the T-cell cap, and synapses [31]. Previous studies in several cell lines indicate that FLOT1 localises to plasma membranes [32-35]. In our study, FLOT1 immunostaining was primarily punctate within the plasma membranes of hEEC and ECC-1 cells and also in the glandular and luminal epithelial cells in human endometrium. This is in agreement with a previous study that identified FLOT1 in punctate structures or microdomains in plasma membranes of COS-7 cells, suggesting the recruitment/localisation of FLOT1 to the membrane compartments [36]. The FLOT1 punctate staining was primarily present apically in glandular and luminal epithelial cells from mid-secretory or receptive phase endometrium. In late-secretory phase endometrium, FLOT1 staining was present basolaterally in the glands and did not appear punctate. This may have been due to the overall lower level of staining seen in late secretory phase compared to mid-secretory phase endometrium, although it did not reach statistical significance. Studies carried out by our group in the past have showed that IL11 localises in the glandular epithelium and that it may be secreted apically into uterine lumen and basally into the endometrium [10]. It is tempting to speculate that the presence of FLOT1 in combination with IL11 facilitates cell adhesion and embryo implantation. Minimal FLOT1 protein was also present in vascular endothelial and smooth muscle cells - the functional significance for this remains to be determined.

## Conclusions

There is a wealth of evidence to support a role for IL11 in endometrial receptivity [37,38]. IL11 regulates endometrial epithelial cell adhesive properties [15] however the mechanisms by which this occurs are poorly defined. This study has identified membrane-associated proteins ANXA2 and FLOT1 as IL11 downstream targets and suggests that IL11 may regulate endometrial epithelial cell adhesion at least in part via these proteins. Currently there are no biochemical markers of endometrial receptivity. This study suggests that FLOT1 may be useful as a marker of receptivity as it is specifically upregulated in the receptive phase. ANXA2 levels in the endometrium have already been associated with receptivity [20]. It however remains to be elucidated whether FLOT1 and ANXA2 are altered in endometrium during the receptive phase of women with subfertility and therefore indicate their utility as markers of receptivity. Further studies on the roles of FLOT1 and ANXA2 in endometrial epithelial cell adhesion are also required to identify whether both factors may be useful as treatment options for endometrial associated subfertility.

This is the first study to demonstrate that proteomics is a powerful approach in identifying plasma membrane proteins that have may have a role in cell adhesion and embryo-endometrial interactions. Our data further defines mechanisms critical for endometrial receptivity and embryo implantation and strengthens the evidence that IL11 is an important regulator of endometrial receptivity. Inadequate adhesion of the blastocyst to the endometrial epithelium results in infertility [38]. Targeting IL11 and its regulated plasma membrane proteins may help define new therapies to enhance or block implantation.

## List of abbreviations

IL11: Interleukin 11; hEEC: human endometrial epithelial cells; ANXA2: annexin A2; FLOT1: flotillin-1; GRP79: 78 kDa glucose-regulated protein; M: membrane; C: cytosol

## Competing interests

The authors declare that they have no competing interests.

## Authors' contributions

JY performed membrane isolation, 2D-DIGE electrophoresis, Western blots, immunohistochemistry, data analysis and assisted in drafting the manuscript. CF and MYL provided technical assistance in 2D-DIGE electrophoresis and assistance in LCMS/MS data analysis. PGS designed and provided assistance in co-ordination of the study and participation in data analysis. ED conceived of the study, designed, participated in data analysis and drafted the manuscript. All authors read and approved the final study.

## Supplementary Material

Additional file 1 **Supplementary Data**. MS/MS analysis information of protein identification. Click here for file
