# Supplementary material for: Proteomic analysis identifies interleukin 11 regulated plasma membrane proteins in human endometrial epithelial cells in vitro
Source: Reprod Biol Endocrinol. 2011 May 30;9:73. doi: 10.1186/1477-7827-9-73 (PMC3130648; doi:10.1186/1477-7827-9-73)
Supplement: Additional file 1 — Supplementary Data. MS/MS analysis information of protein identification. [file 1477-7827-9-73-S1.PDF]

Supplementary Data I

Spot Number	Accession Number	Protein(s) inferred	Number of peptides Identified	Sequence Converage	Position in sequence	Peptide sequence	Precusor m/z	Change Observed	Calculated mass (neutral)	Mascot score	Mascot Expect Value
Spot 9	P45880	sp [P45880] Voltage-dependent anion-selective channel protein 2 Tax_Id= 9609 [Homo sapiens]	5	17.70%	32-39	(K)GFGFGLVK(L)	412.74	2	823.46	29.3	1.26E-001
					178-185	(R)NNFAVGYR(T)	470.74	2	939.46	43.1	6.29E-003
					268-277	(K)LTSALVDGK(S)	508.80	2	1015.59	65.2	5.61E-005
					236-247	(K)YQLDPTASISAK(V)	647.34	2	1292.66	33.8	9.10E-002
					108-121	(K)LTFDTTFSPNTGKK(S)	788.90	2	1555.79	46.9	3.79E-003
	P07355	sp [P07355] Annexin A2 Tax_Id=8606 [Homo sapiens]	13	38.60%	296-302	(R)SEVDMLK(I)	411.21	2	820.40	38.7	2.62E-002
					197 - 204	(R)DLYDAGVK(R)	440.72	2	879.43	39.0	2.04E-002
					50 - 63	(K)GVDEVTIVNILTNR(S)	514.96	3	1541.84	34.6	5.48E-002
					29 - 37	(K)AYTNFDAER(D)	543.75	2	1085.48	52.4	3.62E-004
					38 - 47	(R)DALNIETAIK(T)	544.30	2	1086.59	61.3	1.73E-004
					69 - 77	(R)QDIAFAYQR(R)	556.28	2	1110.54	53.7	6.44E-004
					105 - 115	(K)TPAQYDASELK(A)	611.80	2	1221.59	55.1	7.40E-004
					158 - 168	(K)DIISDTSGDFR(K)	613.29	2	1224.56	52.2	6.15E-004
					158 - 168	(K)DIISDTSGDFR(K)	613.29	2	1224.57	46.7	2.28E-003
					11 - 28	(K)LSLEGDHSTPPSAYGSVK(A)	615.64	3	1843.90	33.0	8.89E-002
					136 - 145	(R)TNQELQEINR(V)	622.82	2	1243.61	60.7	1.38E-004
					153 - 169	(K)TDLEKDIISDTSGDFRK(L)	647.32	3	1938.95	72.0	1.12E-005
					314 - 324	(K)SLYYYIQQDTK(G)	711.35	2	1429.69	66.3	4.16E-005
					82 - 88	(K)ELASALK(S)	731.43	1	730.42	44.0	9.75E-003
					50 - 63	(K)GVDEVTIVNILTNR(S)	771.93	2	1541.84	77.5	2.88E-006
					197 - 204	(R)DLYDAGVK(R)	880.44	1	879.43	34.0	6.44E-002
					11-28	(K)LSLEGDHSTPPSAYGSVK(A)	922.96	2	1843.90	73.2	8.49E-006
	P60891	sp [P60891] Ribose-phosphate pyrophosphokinase 1 Tax_Id=9606 [Homo sapiens]	4	15.10%	205 - 212	(R)MVLVGDVK(D)	430.75	2	859.48	36.8	5.87E-002
					185 - 194	(R)LNVDFALIHK(E)	585.34	2	1168.66	35.7	3.01E-002
					244 - 260	(R)VYAILTHGIFSGPAISR(I)	601.34	3	1800.99	58.4	1.90E-004
					6 - 18	(K)IFSGSSHQDLSQK(I)	717.36	2	1432.70	58.3	2.75E-004
					244 - 260	(R)VYAILTHGIFSGPAISR(I)	901.50	2	1800.99	108.0	2.08E-009

	P52564	sp|P52564|Dual specificity mitogen- activated protein kinase kinase 6 Tax_Id=9606 [Homo	3	11.40%	62 - 69	(R)GAYGVVEK(M)	411.72	2	821.43	37.4	2.94E-002
					179 - 194	(R)DVKPSNVLINALGQVK(M)	565.67	3	1693.98	44.5	1.81E-003
					18 - 31	(K)EAFEQPQTSSTPPR(D)	787.88	2	1573.74	50.4	1.23E-003
					179 - 194	(R)DVKPSNVLINALGQVK(M)	847.99	2	1693.97	52.9	2.88E-004
Spot 14	O75955	sp|O75955|Flotillin-1 Tax_Id=9606 [Homo sapiens]	23	62.30%	404 - 410	(R)LPESVER(L)	415.22	2	828.43	35.2	4.56E-002
					127 - 133	(K)FSEQVFK(V)	442.73	2	883.44	33.9	4.56E-002
					254 - 261	(R)VQVQVVER(A)	478.78	2	955.54	44.9	4.06E-003
					19 - 28	(R)SPPVMVAGGR(V)	485.76	2	969.51	47.6	2.29E-003
					19 - 28	(R)SPPVmVAGGR(V)	493.76	2	985.50	42.3	8.49E-003
					262 - 275	(R)AQQVAVQEQEIARR(E)	542.63	3	1624.87	35.1	5.23E-002
					320 - 330	(R)GEAEAFAIGAR(A)	546.28	2	1090.54	68.0	3.46E-005
					411 - 425	(R)LTGVSISQVNHKPLR(T)	550.32	3	1647.94	36.3	1.54E-002
					393 - 403	(K)VTGEVLDILTR(L)	608.35	2	1214.69	56.6	3.54E-004
					393 - 403	(K)VTGEVLDILTR(L)	608.35	2	1214.69	64.3	6.01E-005
					41 - 51	(R)ISLNTLTLNVK(S)	608.37	2	1214.73	76.2	1.94E-006
					220 - 230	(K)KAAYDIEVNTR(R)	640.34	2	1278.66	71.2	1.48E-005
					134 - 152	(K)VASSDLVNMGISVVSYTLK(D)	661.69	3	1982.04	51.8	1.17E-003
					92 - 109	(K)TEAEIAHIALETLEGHQR(A)	673.35	3	2017.02	65.3	5.48E-005
					92 - 109	(K)TEAEIAHIALETLEGHQR(A)	673.35	3	2017.03	61.4	1.35E-004
					318 - 330	(R)MRGEAEAFAIGAR(A)	689.85	2	1377.68	43.7	8.49E-003
					378 - 392	(K)ITLVSSGSGTMGAAK(V)	690.36	2	1378.71	80.9	1.73E-006
					59 - 72	(R)HGVPISVTGIAQVK(I)	703.41	2	1404.81	44.6	2.88E-003
					232 - 244	(R)AQADLAYQLQVAK(T)	709.89	2	1417.76	99.4	2.34E-008
					199 - 211	(K)VSAQYLSEIEMAK(A)	734.87	2	1467.73	76.1	4.46E-006
					262 - 274	(R)AQQVAVQEQEIAR(R)	735.39	2	1468.76	82.2	1.12E-006
					341 - 360	(K)KAEAFQLYQEAAQLDMLLEK(L)	780.40	3	2338.19	66.9	3.62E-005
					231 - 244	(R)RAQADLAYQLQVAK(T)	787.94	2	1573.86	61.8	9.97E-005
					303 - 317	(K)SQLIMQAEAEAASVR(M)	802.41	2	1602.80	119.0	2.45E-010
					153 - 166	(K)DIHDDQDYLHSLGK(A)	828.39	2	1654.76	61.4	8.11E-005
					361 - 377	(K)LPQVAEEISGPLTSANK(I)	877.47	2	1752.93	85.2	4.56E-007
					134 - 152	(K)VASSDLVNMGISVVSYTLK(D)	992.03	2	1982.04	65.6	5.00E-005

Supplementary Data I

	P06733	sp|P06733|Alpha- enolase Tax_Id=9606 [Homo sapiens]	2	6.68%	270 - 281	(R)YISPDQLADLYK(S)	713.37	2	1424.72	71.2	1.41E-005
					163 - 179	(K)LAMQEFMILPVGAANFR(E)	954.50	2	1906.98	63.2	9.31E-005
	P38646	sp|P38646|Stress-70 protein, mitochondrial Tax_Id=9606 [Homo sapiens]	7	11.50%	301 - 307	(K)DNMALQR(V)	424.21	2	846.40	42.6	8.89E-003
					647 - 653	(K)LFEMAYK(K)	451.23	2	900.44	33.6	4.56E-002
					127 - 135	(R)RYDDPEVQK(D)	575.28	2	1148.55	44.8	5.00E-003
					207 - 218	(K)DAGQISGLNVLR(V)	621.84	2	1241.67	62.0	1.04E-004
					160 - 1733	(K)LYSPSQIGAFVLMK(M)	777.42	2	1552.83	64.2	6.29E-005
					108 - 121	(R)QAVTNPNNTFYATK(R)	784.89	2	1567.76	66.6	3.54E-005
					499 - 513	(K)LLGQFTLIGIPPAPR(G)	796.98	2	1591.94	60.7	2.18E-005
	P28331	sp|P28331|NADH-ubiquinone oxidoreductase 75 kDa subunit, mitochondrial Tax_Id=9606 [Homo sapiens]	8	13.20%	56 - 62	(K)VGMQIPR(F)	400.73	2	799.44	36.0	3.08E-002
					409 - 417	(R)FEAPLFNAR(I)	532.78	2	1063.55	36.1	5.36E-002
					502 - 511	(R)MTSGVTGDWK(V)	541.26	2	1080.50	44.9	3.46E-003
					593 - 602	(K)SATYVNTEGR(A)	549.27	2	1096.51	48.4	1.69E-003
					99 - 108	(K)GWNILTNSEK(S)	581.30	2	1160.58	42.8	1.09E-002
					326 - 336	(R)VAGMLQSFQGK(D)	583.30	2	1164.59	44.5	7.57E-003
					519 - 538	(R)IASQVAALDLGYKPGVEAIR(K)	691.06	3	2070.15	50.7	6.74E-004
					674 - 692	(K)LVNQQLLADPLVPPQLTIK(D)	700.75	3	2099.24	25.7	4.76E-002
					153 - 163	(K)MKETAEAYLGK(K)	414.21	3	1239.62	27.4	3.15E-001
					153 - 163	(K)mKETAEAYLGK(K)	419.55	3	1255.61	30.0	1.41E-001
					123 - 138	(K)KTKPYIQVDIGGGQTK(T)	434.00	4	1731.95	30.8	1.15E-001
					262 - 268	(R)VMEHFIK(L)	452.24	2	902.47	43.3	1.17E-002
					153 - 164	(K)MKETAEAYLGKK(V)	456.91	3	1367.71	54.3	6.44E-004
					153 - 164	(K)mKETAEAYLGKK(V)	462.24	3	1383.71	39.0	2.28E-002
					155 - 163	(K)ETAEAYLGK(K)	491.25	2	980.48	32.6	8.49E-002
					164 - 181	(K)KVTHAVVTVPAYFNDAQR(Q)	504.77	4	2015.06	29.6	1.90E-001
					325 - 336	(R)AKFEELNMDLFR(S)	504.92	3	1511.75	34.4	6.15E-002
					139 - 152	(K)TFAPEEISAMVLTK(M)	512.94	3	1535.79	44.4	7.23E-003
					139 - 152	(K)TFAPEEISAMVLTK(M)	512.94	3	1535.80	53.2	9.31E-004
					47 - 60	(K)NGRVEIIANDQGNR(I)	519.27	3	1554.79	32.7	9.98E-002
					47 - 60	(K)NGRVEIIANDQGNR(I)	519.60	3	1555.77	26.9	3.54E-001
					353 - 367	(K)KSDIDEIVLVGGSTR(I)	530.29	3	1587.85	28.7	2.94E-001

Supplementary Data I

Spot 16	P11021	sp|P11021|78 kDa glucose-regulated protein Tax_Id=9606 [Homo sapiens]	32	47.60%	124 - 138	(K)TKPYIQVDIGGGQTK(T)	535.63	3	1603.86	53.8	9.10E-004
					541 - 554	(R)MVNDAEKFAEEDKK(L)	551.93	3	1652.77	28.6	2.88E-001
					620 - 633	(K)KKELEEIVQPIISK(L)	552.00	2	1652.97	29.0	5.11E-002
					155 - 164	(K)ETAEAYLGKK(V)	555.30	2	1108.58	40.7	1.48E-002
					465 - 474	(K)VYEGERPLTK(D)	596.32	2	1190.63	39.3	2.62E-002
					198 - 214	(R)IINEPTAAAIAYGLDKR(E)	606.00	3	1814.99	35.1	3.97E-002
					186 - 197	(K)DAGTIAGLNVMR(I)	609.32	2	1216.62	96.7	5.12E-008
					448 - 464	(K)SQIFSTASDNQPTVTIK(V)	612.98	3	1835.93	58.3	3.23E-004
					50 - 60	(R)VEIIANDQGNR(I)	614.82	2	1227.62	45.3	4.67E-003
					186 - 197	(K)DAGTIAGLNVmR(I)	617.32	2	1232.62	72.7	1.23E-005
					153 - 163	(K)MKETAEAYLGK(K)	620.82	2	1239.61	65.8	4.35E-005
					153 - 163	(K)mKETAEAYLGK(K)	628.81	2	1255.61	54.5	5.12E-004
					165 - 181	(K)VTHAVVTVPAYFNDAQR(Q)	630.00	2	1886.96	44.7	6.74E-003
					586 - 596	(K)LSSEDKETMEK(A)	648.80	2	1295.59	42.3	6.29E-003
					447 - 464	(K)KSQIFSTASDNQPTVTIK(V)	655.68	3	1964.02	29.9	1.69E-001
					327 - 336	(K)FEELNMDLFR(S)	657.31	2	1312.61	62.5	6.15E-005
					563 - 573	(R)NELESYAYSLK(N)	658.82	2	1315.63	46.2	3.01E-003
					563 - 573	(R)NELESYAYSLK(N)	658.82	2	1315.63	62.9	6.90E-005
					602 - 617	(K)IEWLESHQDADIEDFK(A)	658.98	3	1973.90	43.6	4.25E-003
					164 - 181	(K)KVTHAVVTVPAYFNDAQR(Q)	672.69	3	2015.06	32.0	1.07E-001
					622 - 633	(K)ELEEIVQPIISK(L)	699.40	2	1396.78	65.9	2.94E-005
					102 - 113	(R)TWNDPSVQQDIK(F)	715.85	2	1429.68	65.8	3.79E-005
					634 - 654	(K)LYGSAGPPPTGEEDTAEKDEL(-)	726.00	3	2174.98	52.4	5.12E-004
					139 - 152	(K)TFAPEEISAMVLTK(M)	768.90	2	1535.79	55.6	5.48E-004
					61 - 74	(R)ITPSYVAFTPEGER(L)	783.89	2	1565.77	77.2	4.06E-006
					353 - 367	(K)KSDIDEIVLVGGSTR(I)	794.93	2	1587.85	122.0	1.38E-010
					124 - 138	(K)TKPYIQVDIGGGQTK(T)	802.94	2	1603.86	73.9	8.69E-006
					620 - 633	(K)KKELEEIVQPIISK(L)	827.50	2	1652.97	78.5	5.48E-007
					82 - 96	(K)NQLTSNPENTVFDAK(R)	839.41	2	1676.80	85.2	5.12E-007
					198 - 214	(R)IINEPTAAAIAYGLDKR(E)	908.50	2	1814.99	116.0	3.23E-010
					82 - 97	(K)NQLTSNPENTVFDAKR(L)	839.41	2	1832.90	82.0	1.26E-006
					448 - 464	(K)SQIFSTASDNQPTVTIK(V)	908.50	2	1835.93	98.4	3.08E-008

Supplementary Data I

					475 - 492	(K)DNHLLGTFDLTGIPPAPR(G)	917.46	2	1933.00	81.5	1.38E-006
					602 - 617	(K)IEWLESHQDADIEDFK(A)	918.97	2	1973.90	106.0	2.23E-009
					634 - 654	(K)LYGSAGPPPTGEEDTAEKDEL(-)	1088.50	2	2174.98	100.0	8.89E-009
	Q9Y5P4	sp|Q9Y5P4|Collagen type IV alpha-3-binding protein Tax_Id=9606 [Homo sapiens]	9	17.10%	617 - 624	(K)TAGKPILF(-)	423.76	2	845.50	45.2	5.87E-003
					227 - 234	(K)LFPHVTPK(G)	469.78	2	937.54	35.2	2.56E-002
					161 - 168	(K)LAEMETFR(D)	498.75	2	995.48	54.2	5.00E-004
					161 - 168	(K)LAEmETFR(D)	506.74	2	1011.47	35.1	3.23E-002
					505 - 513	(R)DVLYLSVIR(K)	539.32	2	1076.62	42.9	5.48E-003
					505 - 513	(R)DVLYLSVIR(K)	539.32	2	1076.62	47.6	1.86E-003
					212 - 226	(R)SDGDFLHSTNGNKEK(L)	550.58	3	1648.73	37.3	1.20E-002
					197 - 211	(R)DKVVEDDEDDFPTTR(S)	594.27	3	1779.78	40.9	6.29E-003
					117 - 128	(K)TESGYGSESSLR(R)	636.79	2	1271.56	50.7	5.23E-004
					433 - 445	(R)EVEENGIVLDPLK(A)	727.89	2	1453.77	86.6	3.79E-007
					197 - 211	(R)DKVVEDDEDDFPTTR(S)	890.90	2	1779.78	78.7	1.07E-006
					577 - 595	(K)ITYVANVNPGGWAPASVLR(A)	993.03	2	1984.05	75.2	4.77E-006
	Q15642-2	sp_vs|Q15642-2|(TRIP10)Isoform 2 of Q15642. Tax_Id=9606 [Homo sapiens]	22	50.60%	46 - 354	(K)RLQQQLEER(S)	400.56	3	1198.64	53.6	7.92E-004
					263 - 273	(K)NDSHVLIELHK(S)	435.57	3	1303.69	36.9	4.67E-002
					263 - 273	(K)NDSHVLIELHK(S)	435.90	3	1304.67	38.8	2.88E-002
					34 - 44	(K)ERTEVEQAYAK(Q)	441.89	3	1322.65	32.8	8.89E-002
					254 - 262	(K)VAANAVDPK(N)	442.75	2	883.48	45.6	3.30E-003
					180 - 196	(R)SHMAEESKNEYAAQLQR(F)	498.74	4	1990.92	36.9	2.51E-002
					158 - 166	(R)LDQDINATK(A)	509.27	2	1016.51	58.6	2.94E-004
					347 - 354	(R)LQQQLEER(S)	522.28	2	1042.54	32.3	9.98E-002
					188 - 196	(K)NEYAAQLQR(F)	546.78	2	1091.54	41.8	1.29E-002
					88 - 97	(R)ELVAENLSVR(V)	565.31	2	1128.61	42.5	1.15E-002
					151 - 166	(K)AAQTAERLDQDINATK(A)	582.30	3	1743.88	40.2	2.13E-002
					346 - 354	(K)RLQQQLEER(S)	600.33	2	1198.64	43.6	8.30E-003
					294 - 311	(R)APSDSSLGTPSDGRPELR(G)	614.64	3	1840.89	56.7	3.88E-004
					329 - 344	(K)TVVTEDFSHLPPEQQR(K)	628.32	3	1881.93	46.5	3.97E-003
					430 - 454	(R)HARPPDPPASAPPDSSSNSASQDTK(E)	630.05	4	2516.15	48.5	1.48E-003
					122 - 132	(R)RAQQQLENGFK(Q)	659.85	2	1317.68	37.7	3.88E-002
					34 - 44	(K)ERTEVEQAYAK(Q)	662.33	2	1322.64	33.4	7.74E-002

					34 - 44	(K)ERTEVEQAYAK(Q)	662.33	2	1322.65	73.7	7.39E-006
					408 - 418	(K)YEAWLAEAESR(V)	662.81	2	1323.61	54.7	3.97E-004
					180 - 196	(R)SHMAEESKNEYAAQLQR(F)	664.65	3	1990.92	73.3	5.74E-006
					200 - 215	(R)DQAHFYFSQMPQIFDK(L)	667.98	3	2000.91	32.5	5.87E-002
					17 - 27	(R)HTQWGLDLLDR(Y)	677.35	2	1352.69	58.9	2.34E-004
					529 - 541	(K)EGGEGYVPTSYLR(V)	714.34	2	1426.67	48.8	1.66E-003
					227 - 247	(R)LGAGYGLLSEAELEVVPIIAK(C)	714.74	3	2141.20	59.6	6.74E-005
					67 - 87	(K)FSQQQSFVQILQEVNDFAGQR(E)	823.74	3	2468.21	59.5	2.04E-004
					378 - 400	(K)TPQMGDPASLEPQIAETLSNIER(L)	833.08	3	2496.22	46.5	3.38E-003
					430 - 454	(R)HARPPDPPASAPPDSSSNSASQDTK(E)	839.73	3	2516.15	87.4	1.90E-007
					151 - 166	(K)AAQTAERLDQDINATK(A)	872.94	2	1743.87	79.7	2.34E-006
					294 - 311	(R)APSDSSLGTPSDGRPELR(G)	921.45	2	1840.89	39.5	2.04E-002
					329 - 344	(K)TVVTEDFSHLPPEQQR(K)	941.97	2	1881.92	78.4	2.62E-006
					200 - 222	(R)DQAHFYFSQMPQIFDKLQDMDER(R)	963.77	3	2888.30	46.6	1.77E-003
					227 - 247	(R)LGAGYGLLSEAELEVVPIIAK(C)	1071.61	2	2141.20	72.8	3.38E-006
					67 - 87	(K)FSQQQSFVQILQEVNDFAGQR(E)	1235.11	2	2468.21	108.0	2.94E-009
	P52597	sp|P52597|Heterogeneous nuclear ribonucleoprotein F Tax_Id=9606 [Homo sapiens]	4	19.30%	218 - 224	(R)RYIGIVK(Q)	424.77	2	847.53	9.3	6.59E-002
					99 - 114	(K)HSGPNSADSANDGFVR(L)	544.25	3	1629.72	54.0	2.75E-004
					Feb-14	(M)mLGPEGGEGFVVK(L)	681.34	2	1360.67	52.2	1.20E-003
					151 - 167	(K)ITGEAFVQFASQELAEK(A)	934.48	2	1866.94	90.2	1.90E-007
	P60709	sp|P60709|Actin, cytoplasmic 1 Tax_Id=9606 [Homo sapiens]	9	26.40%	51 - 62	(K)DSYVGDEAQSKR(G)	452.21	3	1353.62	40.7	9.98E-003
					19 - 28	(K)AGFAGDDAPR(A)	488.73	2	975.44	61.0	6.90E-005
					184 - 191	(R)DLTDYLMK(I)	499.75	2	997.48	36.0	3.01E-002
					85 - 95	(K)IWHHTFYNELR(V)	505.92	3	1514.74	38.7	2.34E-002
					360 - 372	(K)QEYDESGPSIVHR(K)	506.24	3	1515.70	49.2	1.35E-003
					316 - 326	(K)EITALAPSTMK(I)	581.31	2	1160.61	36.5	5.36E-002
					51 - 61	(K)DSYVGDEAQSK(R)	599.77	2	1197.51	43.0	2.81E-003
					51 - 61	(K)DSYVGDEAQSK(R)	599.77	2	1197.51	62.9	2.88E-005
					96 - 113	(R)VAPEEHPVLLTEAPLNPK(A)	652.03	3	1953.06	35.3	3.54E-002
					51 - 62	(K)DSYVGDEAQSKR(G)	677.82	2	1353.62	45.5	3.30E-003
					239 - 254	(K)SYELPDGQVITIGNER(F)	895.95	2	1789.80	86.3	5.00E-007
		sp|P50502|Hsc70-			33 - 41	(R)EWVESMGGK(V)	511.74	2	1021.45	39.2	1.07E-002

	P50502	interacting protein Tax_Id=9606 [Homo sapiens]	4	12.50%	211 - 222	(K)LDYDEDASAMLK(E)	685.81	2	1369.61	85.9	1.77E-007
					18 - 29	(K)QDPSVLHTEEMR(F)	721.34	2	1440.66	37.5	1.99E-002
					174-186	(R)AIEINPDSAQPYK(W)	723.37	2	1444.72	58.1	2.81E-004
	P05787	sp|P05787|Keratin, type II cytoskeletal 8 Tax_Id=9606 [Homo sapiens]	25	46.80%	102 - 108	(K)FASFIDK(V)	414.22	2	826.42	41.7	7.74E-003
					394 - 401	(K)LLEGEESR(L)	466.74	2	931.46	49.5	2.08E-003
					274 - 285	(R)SRAEAESMYQIK(Y)	471.57	3	1411.68	32.1	7.92E-002
					305 - 312	(K)TEISEMNR(N)	490.23	2	978.44	43.0	5.87E-003
					402 - 414	(R)LESGMQNMSIHTK(T)	492.57	3	1474.69	33.2	6.01E-002
					187 - 198	(R)TEMENEFVLIKK(D)	494.26	3	1479.77	40.2	2.04E-002
					317 - 325	(R)LQAEIEGLK(G)	500.79	2	999.56	47.6	3.30E-003
					123 - 130	(K)WSLLQQQK(T)	515.79	2	1029.56	33.9	1.02E-001
					393 - 401	(R)KLLEGEESR(L)	530.79	2	1059.56	57.1	5.24E-004
					265 - 273	(K)AQYEDIANR(S)	540.26	2	1078.50	47.4	2.45E-003
					102 - 110	(K)FASFIDKVR(F)	541.80	2	1081.59	61.0	1.09E-004
					353 - 362	(K)LSELEAALQR(A)	565.31	2	1128.61	90.7	1.82E-007
					276 - 285	(R)AEAESMYQIK(Y)	585.28	2	1168.54	31.3	9.31E-002
					473 - 483	(K)LVSESSDVLPK(-)	587.32	2	1172.63	39.6	2.45E-002
					199 - 213	(K)DVDEAYMNKVELESR(L)	599.95	3	1796.82	41.4	7.74E-003
					303 - 312	(R)TKTEISEMNR(N)	604.80	2	1207.59	59.0	2.39E-004
					177 - 186	(K)NKYEDEINKR(T)	654.83	2	1307.65	39.1	2.51E-002
					253 - 264	(R)SLDMDSIIAEVK(A)	660.84	2	1319.66	90.1	2.18E-007
					329 - 341	(R)ASLEAAIADAEQR(G)	672.84	2	1343.67	79.1	2.18E-006
					187 - 197	(R)TEMENEFVLIK(K)	676.84	2	1351.67	39.1	2.34E-002
					159 - 176	(K)LKLEAELGNMQGLVEDFK(N)	678.69	3	2033.05	42.9	9.31E-003
					214 - 225	(R)LEGLTDEINFLR(Q)	710.38	2	1418.74	61.4	1.66E-004
					102 - 108	(K)FASFIDK(V)	827.43	1	826.42	36.3	2.56E-002
					161 - 176	(K)LEAELGNMQGLVEDFK(N)	896.94	2	1791.87	70.3	1.66E-005
					134 - 148	(R)SNMDNMFESYINNLR(R)	924.41	2	1846.80	111.0	5.12E-010
					234 - 252	(R)ELQSQISDTSVVLSMDNSR(S)	1055.01	2	2108.01	123.0	7.92E-011
					395 - 403	(R)GYPTLLLFR(G)	540.32	2	1078.62	36.2	2.56E-002
					395 - 403	(R)GYPTLLLFR(G)	540.32	2	1078.62	48.5	1.51E-003
					395 - 403	(R)GYPTLLLFR(G)	540.32	2	1078.62	36.4	2.18E-002

Supplementary Data I

Spot 22	Q8NBS9	sp|Q8NBS9|Thioredoxin domain-containing protein 5 Tax_Id=9606 [Homo sapiens]	11	27.30%	395 - 403	(R)GYPTLLLFR(G)	540.32	2	1078.62	34.2	3.62E-002
					395 - 403	(R)GYPTLLLFR(G)	540.32	2	1078.62	39.7	9.31E-003
					423 - 432	(R)FVLSQAKDEL(-)	575.31	2	1148.61	41.8	1.38E-002
					288 - 296	(R)EYVESQLQR(T)	576.29	2	1150.56	51.1	1.38E-003
					261 - 269	(R)GYPTLLWFR(D)	576.81	2	1151.61	40.8	1.55E-002
					355 - 365	(K)TLAPTWEELSK(K)	637.84	2	1273.66	58.8	3.23E-004
					95 - 105	(R)LQPTWNDLGDK(Y)	643.82	2	1285.63	32.2	9.31E-002
					189 - 211	(K)QGLYELSASNFELHVAQGDHFIK(F)	651.58	4	2602.29	48.7	2.13E-003
					355 - 366	(K)TLAPTWEELSKK(E)	701.88	2	1401.75	62.9	1.15E-004
					282 - 287	(R)DLESLR(E)	732.39	1	731.38	36.7	5.12E-002
					367 - 375	(K)EFPGLAGVK(I)	917.51	1	916.50	41.1	2.18E-002
					222 - 241	(K)ALAPTWEQLALGLEHSETVK(I)	1097.08	2	2192.15	78.6	2.29E-006
	P43686	sp|P43686|26S protease regulatory subunit 6B Tax_Id=9606 [Homo sapiens]	9	22.70%	115 - 121	(R)ILSTIDR(E)	409.24	2	816.47	34.0	8.89E-002
					218 - 229	(K)AVAHHTTAAFIR(V)	432.24	3	1293.70	31.2	1.31E-001
					314 - 326	(R)ADTLDPALLRPGR(L)	465.60	3	1393.77	30.5	1.17E-001
					275 - 283	(R)FDAQTGADR(E)	490.73	2	979.44	55.8	2.04E-004
					230 - 238	(R)VVGSEFVQK(Y)	496.77	2	991.53	39.7	1.99E-002
					274 - 283	(K)RFDAQTGADR(E)	568.78	2	1135.54	44.7	5.11E-003
					1-Oct	(-)mEEIGILVEK(A)	601.82	2	1201.63	66.3	5.12E-005
					179 - 192	(R)EAVELPLTHFELYK(Q)	844.95	2	1687.88	54.4	7.74E-004
					179 - 192	(R)EAVELPLTHFELYK(Q)	844.95	2	1687.88	57.1	4.06E-004
					47 - 66	(K)LQQELEFLEVQEEYIKDEQK(N)	846.76	3	2537.26	46.5	3.88E-003
	P60842	sp|P60842|Eukaryotic initiation factor 4A-I Tax_Id=9606 [Homo sapiens]	5	16.00%	162 - 168	(R)VFDMLNR(R)	447.73	2	893.44	33.9	7.23E-002
					147 - 161	(K)LQMEAPHIIVGTPGR(V)	540.30	3	1617.87	52.3	1.09E-003
					46 - 61	(R)GIYAYGFEKPSAIQQR(A)	609.98	3	1826.93	30.8	1.48E-001
					69 - 82	(K)GYDVIAQAQSGTGK(T)	697.85	2	1393.68	120.0	1.99E-010
					178 - 190	(K)MFVLDEADEMLSR(G)	778.36	2	1554.71	93.7	5.00E-008
					46 - 56	(R)VTHELQAMKDK(I)	433.90	3	1298.67	31.1	1.31E-001
					349 - 355	(R)IMQIHSR(K)	442.74	2	883.47	47.6	2.18E-003
					46 - 54	(R)VTHELQAMK(D)	528.78	2	1055.54	50.2	1.41E-003
					20 - 28	(K)MSTEEIIQR(T)	553.78	2	1105.54	59.7	2.29E-004
					319 - 328	(R)VDILDPALLR(S)	562.84	2	1123.66	43.7	2.75E-003

Supplementary Data I

	A8K781	tr|A8K781|cDNA FLJ75299, highly similar to Xenopus laevis proteasome (prosome, macropain) 26S subunit, ATPase 3, mRNA Tax_Id=9606 [Homo sapiens]	17	47.00%	114 - 128	(R)QTYFLPVIGLVDAEK(L)	564.98	3	1691.92	49.3	1.73E-003
					206 - 217	(K)GVLMYGPPGTGK(T)	588.81	2	1175.60	49.8	2.45E-003
					178 - 193	(K)QIQELVEAIVLPMNHK(E)	621.68	3	1862.01	40.2	1.20E-002
					261 - 278	(K)EKAPSIIFIDELDAIGTK(R)	654.03	3	1959.06	55.5	3.79E-004
					2-19	(M)aTVWDEAEQDGIGEEVLK(M)	677.66	3	2029.95	31.7	9.31E-002
					194 - 205	(K)EKFENLGIQPPK(G)	700.38	2	1398.75	75.0	5.48E-006
					335 - 346	(R)KIEFPMPNEEAR(A)	730.87	2	1459.72	52.2	1.23E-003
					158 - 177	(K)AMEVDERPTEQYSDIGGLDK(Q)	751.68	3	2252.03	64.6	3.97E-005
					235 - 250	(K)LAGPQLVQMFIGDGAK(L)	822.94	2	1643.87	81.9	1.29E-006
					417 - 423	(K)ANLQYYA(-)	842.41	1	841.40	35.4	1.90E-002
					114 - 128	(R)QTYFLPVIGLVDAEK(L)	846.96	2	1691.91	69.0	1.95E-005
					263 - 278	(K)APSIIFIDELDAIGTK(R)	851.97	2	1701.92	62.1	1.07E-004
					178 - 193	(K)QIQELVEAIVLPMNHK(E)	931.52	2	1861.02	55.9	2.94E-004
					140 - 155	(K)DSYLILETLPTEYDSR(V)	957.97	2	1913.93	36.8	3.79E-002
					2 - 19	(M)aTVWDEAEQDGIGEEVLK(M)	1015.98	2	2029.94	77.1	2.51E-006
	P53990	sp|P53990|Uncharacterized protein KIAA0174 Tax_Id=9606 [Homo sapiens]	5	14.60%	165 - 171	(R)YLIEIAK(N)	425.26	2	848.50	29.4	1.07E-001
					83 - 90	(R)FGLIQSMK(E)	462.26	2	922.49	48.5	2.23E-003
					110 - 118	(R)LQSEVAELK(I)	508.79	2	1015.55	44.4	9.75E-003
					138 - 147	(R)TNQIGTVNDR(L)	559.28	2	1116.55	59.9	1.69E-004
					91 - 109	(K)ELDSGLAESVSTLIWAAPR(L)	672.35	3	2014.04	37.4	3.46E-002
					91 - 109	(K)ELDSGLAESVSTLIWAAPR(L)	1008.03	2	2014.04	60.2	1.86E-004

Table Explanations:	
Spot No.	This is the sample number given to your sample.
Protein(s) inferred	This is the list of inferred proteins found in your sample.
Number of Peptides Identified	Total number of unique peptides sequenced by MS/MS analysis from each protein.
% Coverage	This value is derived from the total number of amino acids sequenced divided by the full protein sequence.
Accession Number	This is the database accession number that is assigned to the inferred protein for sequence retrieval.

Supplementary Data I

Information on MS/MS database search	
Peaklist generating software	extract_msn (Version 2.0, Thermo Fisher Scientific)
Parameters used	Minimum mass m/z 700, Maximum mass m/z 5000.<br>Grouping tolerance, 1.5.<br>Intermediate scans, 1.<br>Minimum scans per group, 1.<br>Precursor charge, AUTO<br>Minimum peaks in .DTA, 10.
Search engine	Mascot algorithm (Version 2.2.01, Matrix Science)
**Search Parameters**	
Enzyme specificity considered	Trypsin
# of missed cleavages permitted	3. Considers partial fragments where the digest was not complete.
Fixed Modifications	+58 Da for Carboxymethyl (Cysteine) if iodoacetic acid was used for the alkylation process.<br>+57 Da for Carbamidomethyl (Cysteine) if iodoacetamide was used for the alkylation process.
Variable Modifications	+42 Da for Acetylation (N-terminus) and +16 Da for Oxidation (Methionine).
Mass tolerance for precursor ions	For LC-ESI Ion Trap MS data, +/- 1.5 Da.
Mass tolerance for fragment ions	For LC-ESI Ion Trap MS/MS data, +/- 0.6 Da.
Database searched	LudwigNR (Version Q1 2009). The latest protein nonidentical database produced by Ludwig Institute for Cancer Research
Species restriction	None - unless specified by User.
# protein entries actually searched	All LudwigNR proteins (i.e., currently over 8 million proteins).
Cut-off score for accepting individual MS/MS spectra	All peptide identifications are validated irrespective of thresholds.
**Protein appears in database under different names and accession numbers**	
If peptides match to multiple proteins, criteria used for selecting which one to report	Where possible, SwissProt database proteins are reported as they provide the most annotation. If multiple SwissProt entries match, then a note is made in brackets after the protein name. If a SwissProt database protein does not exist then only one matching protein is selected for reporting (regardless of the number of homologues). If you are interested in homology
